# Immunogenicity Analysis of Chikungunya Virus DNA Vaccine Based on Mutated Putative N-Linked Glycosylation Sites of the Envelope Protein

**DOI:** 10.3390/vaccines12101097

**Published:** 2024-09-26

**Authors:** Kwangwook Kim, Seo Young Moon, Seungyeon Kim, In-Ohk Ouh, Yookyoung Lee, Heeji Lim

**Affiliations:** Division of Vaccine Development Coordination, Center for Vaccine Research National Institute of Infectious Diseases, Korea National Institute of Health, Korea Disease Control and Prevention Agency, Cheongju-si 28159, Chungcheongbuk-do, Republic of Korea; kwkim83@korea.kr (K.K.); msy1477@korea.kr (S.Y.M.); hatmddus135@korea.kr (S.K.); dvmoio@korea.kr (I.-O.O.); leeykyoung@korea.kr (Y.L.)

**Keywords:** chikungunya, vaccine

## Abstract

Chikungunya fever is a mosquito-borne infectious disease caused by the chikungunya virus (CHIKV). Recently, CHIKV has spread rapidly worldwide, raising global concerns. However, there is only one approved vaccine is available to prevent CHIKV infection; therefore, different platform vaccines development is a public health priority. The CHIKV genome encodes four non-structural polyproteins (nsP1-4) and one structural polyprotein (capsid, envelope 3, envelope 2, 6 K, and envelope 1). Previous studies have shown that N-linked glycans in viral proteins play important roles in regulating immune responses. Accordingly, in this study, we designed four CHIKV DNA vaccine candidates with mutated N-glycosylation sites in the full-length E and E I/II proteins. Our results indicated that immunization of mice with the vaccine elevated the cytokines levels, including IFN-γ, associated with T cell immune response. Furthermore, the truncated E protein with a deleted E III domain (E I/II) exhibited better immunogenicity than the full-length E protein, and N-linked glycosylation of E I/II protein induced a higher cell-mediated immune response. Overall, our study demonstrates that N-linked glycosylation of the E I/II proteins of CHIKV significantly enhances cell-mediated immune responses, laying the foundation for the development of potential vaccination strategies against CHIKV.

## 1. Introduction

In recent years, chikungunya virus (CHIKV) infection has raised public health concerns owing to the increasing number of cases worldwide and the associated co-morbidities. CHIKV was first discovered in Tanzania in 1952 [1], and it had sporadic outbreaks in Africa and Asia, spreading rapidly worldwide in 2004, causing massive outbreaks [2,3,4]. CHIKV is a non-fatal, self-limiting infection characterized by fever, nausea, rashes [5], and polyarthralgia that can persist for years [6]. It is a biosafety level 3 virus and one of the deadliest pathogens, causing chikungunya fever when bitten by mosquitoes infected with the virus. Currently, there is no effective treatment for chikungunya and only one vaccine, a live attenuated vaccine, has been approved; it is a major pathogen that requires extensive research to prepare for epidemics due to global warming. CHIKV is an enveloped, positive-sense, single-stranded RNA virus belonging to the Togaviridae family of alphaviruses. CHIKV encodes both structural (C-E3-E2-6K-E1) and non-structural (nsP1, helicase nsP2, nsP3, and polymerase nsP4) proteins [7,8,9]. The CHIKV envelope is composed of glycoproteins E1 and E2 arranged in a T4 icosahedral symmetry [10,11]. Structural E1–E2 protein heterodimers, the main components of the virus surface [12], mediate fusion and binding to the target cell membrane [13]. N-linked glycosylation is one of the most common post-translational modifications of secretory and membrane-associated proteins. In this process, a high mannose core is added to the side-chain group of an asparagine (N) residue present in the conserved glycosylation motif N-X-S/T [14,15]. Several studies have shown that N-linked glycosylation facilitates the proper folding and trafficking of cellular and viral proteins [16]. N-glycosylation of viral proteins has been shown to influence infection by a variety of viruses, promote viral interactions with cellular receptors, and alter virus recognition by the host immune system by modifying protein immunogenicity [16]. In the case of Sindvivirus (SINV) of the alphavirus family, N-glycosylation of E1 and E2 is essential for viral infection. These experiments revealed a marked decrease in infectivity and replication efficiency in glycosylation-deficient mutants, highlighting the importance of this modification for viral fitness [17]. In addition, studies have been published using Cryo-EM to analyze the structure and N-linked glycosylation sites in alphaviruses, thus providing important data to aid comprehension of alphavirus infection and pathogenicity, and contributing to vaccines and treatment development [18,19]. Research on N-glycosylation patterns of alphaviruses continues to provide insights into the molecular mechanisms of viral infection and host interactions, leading to potentially improved strategies for preventing and treating alphavirus-related diseases. The CHIKV E1 protein has one N-linked glycosylation site at N141, and the E2 protein has two glycosylation sites at N263 and N345. In the present study, we designed four DNA-based vaccine candidates expressing different domains of the CHIKV E protein with or without N-glycosylation and investigated the humoral and cellular immune responses they evoke in mice. Overall, we confirmed an effective cell-mediated immune response to a DNA vaccine candidate in C57BL/6 mice.

## 2. Materials and Methods

### 2.1. Cells, Viruses, and Animals

Vero E6 cells were cultured in complete medium (Dulbecco’s Modified Eagle’s Medium; DMEM, Gibco^TM^, Thermo Fisher Scientific, Waltham, MA, USA) containing 10% heat-inactivated fetal bovine serum (FBS, Gibco^TM^), 2 mM L-glutamine, 1 mM sodium pyruvate, 10,000 U/mL penicillin, and 10,000 μg/mL streptomycin. Cells were incubated in a 5% CO_2_ humidified incubator at 37 °C. The CHIKV strain (NCCP 43132) used in this study was collected from human isolates in Korea and was provided by the National Culture Collection for Pathogens (NCCP). CHIKV strains were passaged and titrated as plaque-forming units (PFU) in Vero E6 cells. All experiments, including those with live viruses, were conducted in a biosafety level 3 (BSL-3) facility following the recommended safety precautions and measures. Four- to five-week-old female C57BL/6 mice were used for the experiments. This study was approved by the Institutional Animal Care and Use Committee (IACUC) of the Korea Disease Control and Prevention Agency (KDCA-IACUC-23-003).

### 2.2. Construction of DNA-Based CHIKV Vaccine

The full-length gene sequence encoding the E protein (C-E3-E2-6K-E1, 1–1248 nucleotides) of the CHIKV strain (NCCP 43132) was optimized using the Optimum Gene™ algorithm to enhance its expression and synthesized by GenScript Biotech (Piscataway, NJ, USA). The synthetic full-length E, E I/II, EΔGln (mutation at N141 and 263 sites), and EI/IIΔGln (mutation at N141 and 263 sites) proteins were digested with BamHI and XhoI and subcloned into the mammalian expression vector pVax1 (Invitrogen^TM^, Thermo Fisher Scientific). The recombinant plasmid was purified using EndoFree Plasmid Giga Kit (QIAGEN GmbH, Hilden, Germany).

### 2.3. Immunization of Mice

C57BL/6 mice were immunized twice or thrice time with 100 μg of the DNA vaccine candidate based on diverse genotypes at 3-week intervals. After the anesthetized mice were injected with a single dose of DNA vaccine, a two-needle array electrode pair was immediately inserted into the tibialis anterior muscle. Three pulses of 100 V (50 ms duration) were delivered using an ECM 830 square-wave electrophoresis system (BTX, Holliston, MA, USA). To evaluate humoral immune responses, blood samples were collected 4 and 7 weeks after the first vaccine dose was administered.

### 2.4. IgG Level Measurement by Enzyme-Linked Immunosorbent Assay (ELISA)

Nunc MaxiSorp 96-well plates (Thermo Fisher Scientific, Waltham, MA, USA) were coated with 100 ng/well of CHIKV E2 protein (SinoBiological, Beijing, China) and incubated overnight at 4 °C. The protein was then removed, and the wells were washed thrice with phosphate-buffered saline (PBS) containing 0.05% Tween-20 (0.05% PBS-T) and blocked with PBS containing 1% bovine serum albumin (BSA) for 2 h at 37 °C. After washing thrice with 0.05% PBS-T, the immunized mouse sera (initial dilution 1:100) were serially diluted 2-fold, added to the CHIKV E2 protein-coated plates, and incubated for 1 h at 37 °C. After washing thrice with 0.05% PBS-T, horseradish peroxidase (HRP)-conjugated anti-mouse IgG (Invitrogen, USA) was added to the wells and incubated at 37 °C for 1 h. Then, the wells were washed five times with 0.05% PBS-T, and tetramethylbenzidine (TMB) substrate (Thermo Fisher Scientific, USA) was added and incubated for 10 min at RT. The reaction was stopped by adding the corresponding stop solution of TMB substrate (Thermo Fisher Scientific, USA), and the absorbance was measured at 450 nm using a microplate reader (Spectra Max i3X, Molecular Devices, San Jose, CA, USA).

### 2.5. CHIKV Neutralization Assay

The plaque reduction neutralization test (PRNT) was performed to evaluate viral neutralization. Vero cells were seeded in 12-well plates (2.5 × 10^5^ cells/well) and incubated overnight at 37 °C in a 5% CO_2_ incubator. CHIKV at 60 plaque-forming units (PFU) were mixed with an equal volume of 2-fold serial dilutions of heat-inactivated immunized mouse serums and incubated at 37 °C for 1 h. Then, the serum-virus mixtures were used to infect Vero E6 cells in the prepared 12-well plates. After 1 h incubation, the infected cells were then overlaid with 1% agarose (Lonza, Basel, Switzerland) in 2× Minimum Essential Medium (Gibco, USA) containing 4% heat-inactivated FBS (Gibco^TM^, USA) and incubated for 3 days at 37 °C in a 5% CO_2_ incubator. Following incubation, the cells were fixed with 10% formaldehyde and stained with 0.8% crystal violet solution. The neutralizing antibody titer was defined as the dilution factor corresponding to 50% plaque reduction compared with the positive control (virus only). Based on the Kärber formula, Neutralizing antibody titer (NT titer) was calculated for the PRNT titer [20].

### 2.6. Cytokine (IFN-γ) Expression Analysis

Enzyme-linked immunosorbent spot (ELISpot) assay for interferon-gamma (IFN-γ)-secreting splenocytes was performed using the mouse IFN-γ ELISpot commercial kit (R&D Systems, Minneapolis, MN, USA) following the manufacturer’s protocol. Splenocytes (5 × 10^5^ cells/well) from immunized mice were seeded in ELISpot 96-well plates (Millipore, Burlington, MA, USA) and cultured in RPMI1640 medium (Gibco^TM^, USA) stimulated with 1 μg of a CHIKV E1, E2, E3, or 6 K protein peptide pool overnight at 37 °C. The plates were washed with wash buffer, and biotinylated anti-IFN-γ antibody was added and incubated at RT for 2 h. Then, alkaline phosphatase-conjugated streptavidin was added to each well and incubated at RT for 2 h. Finally, 3-amino-9-ethylcarbazole chromogen (ACE) solution was added at RT for 20 min. The development of colored spots was monitored and counted using a CTL Immunospot reader (Cleveland, OH, USA). Cytokine expression was examined using the MILLIPLEX Mouse High Sensitivity T Cell Magnetic Bead Panel kit (Millipore, Burlington, MA, USA) according to the manufacturer’s instructions, and the data was analyzed using the MAGPIX system (Luminex Corp., Austin, TX, USA).

### 2.7. Multiplex Cytokine Measurement by ELISA

Culture supernatants from the immunized mouse splenocytes were harvested at specific time points and stored at −80 °C. The levels of IFN-γ and IL-6 were determined by ELISA using a commercial kit (R&D Systems, Minneapolis, MN, USA) according to the manufacturer’s instructions. Cytokine concentrations were calculated using standard curves generated from recombinant cytokines.

### 2.8. Statistical Analysis

The level of significance in the differences between groups was determined by one-way analysis of variance (ANOVA), followed by Tukey’s multiple comparison test using GraphPad Prism 9 software (San Diego, CA, USA). Data with probability values of * *p* < 0.05, ** *p* < 0.005, *** *p* < 0.0005, and **** *p* < 0.0001 were considered statistically significant.

## 3. Results

### 3.1. Designing of DNA Vaccine Candidates against Chikungunya Virus

**Figure 1:**
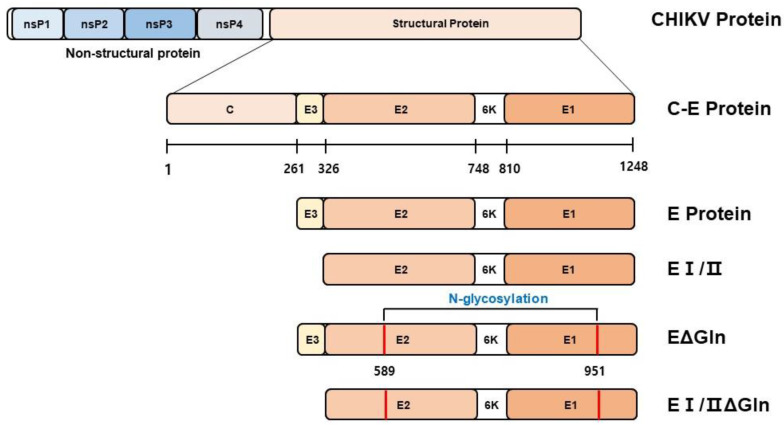
Design of the candidate Chikungunya virus (CHIKV) DNA vaccines. Schematic representation of the DNA vaccine constructs. Four constructs were generated. One construct expressed the full-length E protein, whereas the others expressed a truncated E protein by deleting the E3 domain (E I/II). The positions of the N-linked glycosylation mutations are indicated by solid red bars on the E protein gene.

We constructed four candidate DNA vaccines against chikungunya virus (CHIKV) with mutations in the estimated N-glycosylation sites in the full-length envelope (E) and E I/II proteins (Figure 1). The N-X-S/T glycan motif was abrogated by the synthesis of new genes. The furin cleavage site was removed from the optimized DNA sequence of the CHIKV E protein to enhance its expression. CHIKV E and E I/II proteins were established by replacing asparagine (N) with alanine (A). The optimized DNA sequence was digested with BamHI and XhoI and then cloned into the expression vector pVax1.

### 3.2. Humoral Immune Response Evoked in Mice by the Candidate Vaccines

**Figure 2:**
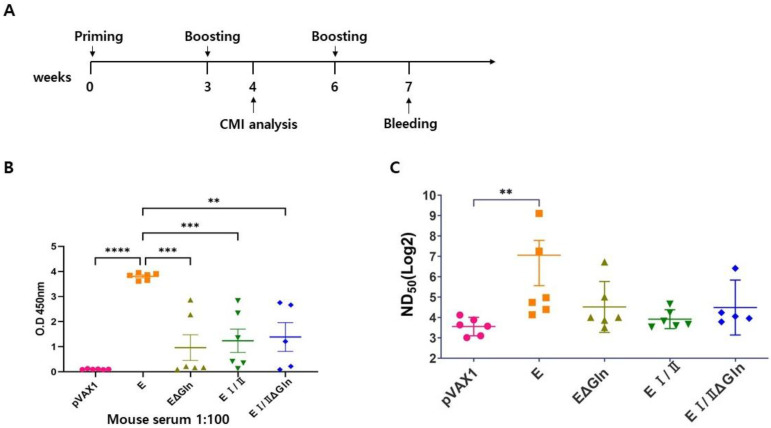
Humoral immune response elicited by various CHIKV E protein DNA vaccines. (**A**) Immunization and analysis timeline of the present study. C57BL/6 mice (*n* = 5–6) received 100 μg of DNA vaccine at weeks 0, 3, and 6, with blood samples collected at week 7 (one week after the second booster). (**B**) Sera from individual mice were serially diluted, and anti-CHIKV-E2 protein specific total IgG levels were quantified using ELISA. (**C**) NAb responses were measured using a viral neutralization assay, with titers represented as the serum dilution required to achieve PRNT50 of viral infection in vero cells. Data represent the mean ± SEM for individual mice in each group. *p*-values were calculated using one-way ANOVA followed by the Kruskal-Wallis test. ** *p* < 0.005, *** *p* < 0.0005, **** *p* < 0.0001. CHIKV, Chikungunya virus; Nab, neutralizing antibody; PRNT, plaque reduction neutralization test; ELISA, enzyme-linked immunosorbent assay; ANOVA, analysis of variance.

To evaluate the humoral immune response evoked by the vaccine candidates, we immunized the mice thrice at 3-week intervals with the DNA vaccines of the CHIKV envelop (E), N-linked glycan mutant E (EΔGln), E I/II, and N-linked glycan mutant E I/II (EI/IIΔGln) proteins in mice (*n* = 5–6), as shown in Figure 2A. Mouse sera were collected one week after the final vaccine administration to analyze CHIKV-specific immunoglobulin G (IgG) levels via ELISA and neutralizing antibody responses. ELISA analysis of the collected mouse serum showed that the levels of CHIKV-specific total IgG antibodies in the serum of mice immunized with the E protein were higher than those in the other groups. In addition, to confirm whether the serum antibodies could neutralize CHIKV infection in vitro, PRNT was performed using serially diluted serum samples. The PRNT analysis, similar to the ELISA results, showed that IgG levels were higher in the full-length E protein DNA vaccine group than in the truncated EΔGln, E I/II ΔGln, or E I/II DNA vaccine groups (Figure 2B). Accordingly, when humoral immune responses in vaccinated mice were evaluated, the mice immunized with the full-length E protein DNA vaccine candidate exhibited the highest levels of cytokines and neutralizing antibody titers. Our results indicated that the E I/II protein or N-linked glycan mutant E proteins induced lower humoral immune responses than the full-length E protein.

### 3.3. Cell-Mediated Immune Response Elicited by DNA Vaccines against CHIKV Based on N-Linked Glycan Mutants of the E Protein

**Figure 3:**
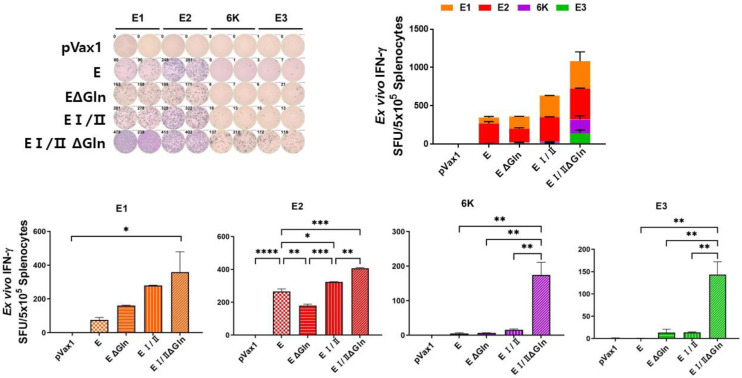
ELISPOT by the different CHIKV E protein DNA vaccines. C57BL/6 mice (n = 6) were immunized with 100 μg DNA vaccine at weeks 0, 3, and 6. Immunized mice were sacrificed 1 week after the First boost, and splenocytes were isolated for measuring ELISPOT assay. Splenocytes (5 × 10^5^) from mice were stimulated for 18 h with 1 μg CHIKV E protein peptide pool. IFN-γ ELISPOT was then performed, and the spots were counted using the CTL Immunospot reader. Data are the mean with SEM of mice within each group. *p*-values were determined using Two-way ANOVA followed by Tukey’s post-hoc test. * *p* < 0.05, ** *p* < 0.005, *** *p* < 0.0005, **** *p* < 0.0001.

**Figure 4:**
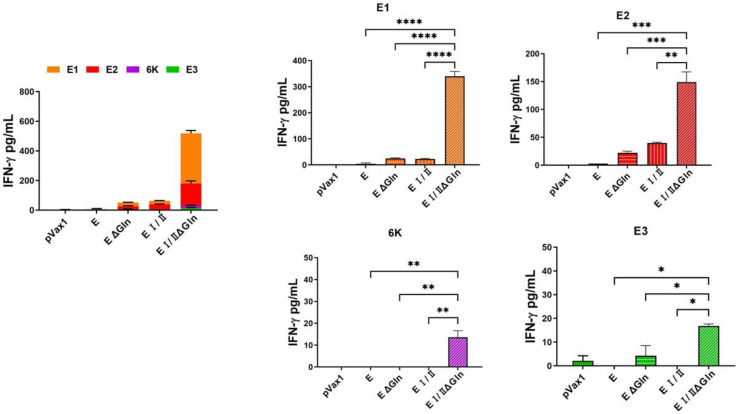
Multiplex assay for the CHIKV E protein-based DNA vaccines. C57BL/6 mice (n = 6) were immunized with 100 μg DNA vaccine at weeks 0, 3, and 6. Immunized mice were sacrificed 1 week after the first boost, and splenocytes were isolated for measuring multiplex assay. Splenocytes (5 × 10^5^) from mice were stimulated for 18 h with 1 μg CHIKV E protein peptide pool. IFN-γ cytokine levels were measured in a mouse cytokine multiplex assay using the culture supernatants of mice splenocytes. *p*-values were determined using Two-way ANOVA followed by Tukey’s post-hoc test. * *p* < 0.05, ** *p* < 0.005, *** *p* < 0.0005, **** *p* < 0.0001.

**Figure 5:**
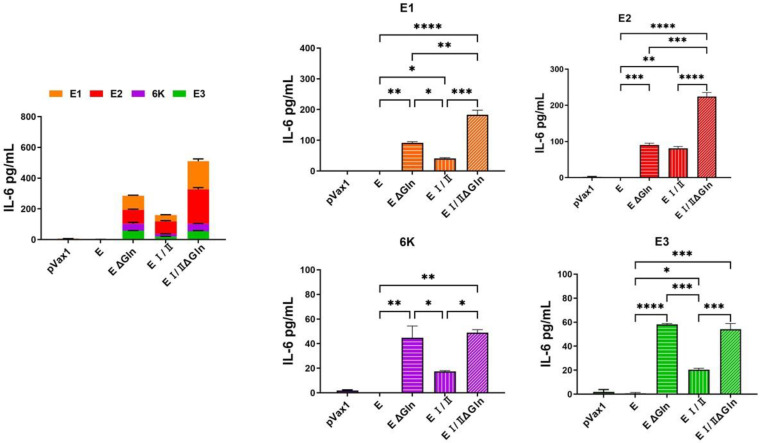
Multiplex assay by the different CHIKV E protein DNA vaccines. C57BL/6 mice (n = 6) were immunized with 100 μg DNA vaccine at weeks 0, 3, and 6. Immunized mice were sacrificed 1 week after the first boost, and splenocytes were isolated for measuring multiplex assay. Splenocytes (5 × 10^5^) from mice were stimulated for 18 h with 1 μg CHIKV E protein peptide pool. IL-6 cytokine level was measured in a mouse cytokine multiplex assay using the culture supernatants of mice splenocytes. *p*-values were determined using Two-way ANOVA followed by Tukey’s post-hoc test. * *p* < 0.05, ** *p* < 0.005, *** *p* < 0.0005, **** *p* < 0.0001.

We also performed an IFN-λ ELISpot assay using vaccinated mouse splenocytes stimulated with CHIKV E peptide pool (E1, E2, E3, and 6 K) and a cytokine multiplex assay to measure the level of antigen-specific T cell response induced by the DNA vaccine candidates. The ELISpot assay showed that the level of IFN-λ in the E I/IIΔGln group was elevated compared to the other groups (Figure 3). Additionally, the results of the cytokine multiplex assay were similar to those of the ELISpot assay. The level of IFN-λ (Figure 4) and interleukin-6 (IL-6; Figure 5) in the E I/IIΔGln group was higher than that in the E, EΔGln, and EI/II groups. Our results indicated that the N-linked glycosylated EI/II protein induced a higher cell-mediated immune response than the full-length E protein.

## 4. Discussion

Chikungunya virus (CHIKV) is one of the priority pathogens selected by the CEPI, with a focus on expanding treatment access to vulnerable populations in endemic countries, and is known to have a high potential to cause future pandemics [21]. The fatality rate of CHIKV infection is reported to be as 5%, and it mainly affects the elderly, neonates, young children, and patients with co-morbidities [5].

Recently, Valneva’s single-dose live-attenuated Ixchiq (VLA1553) vaccine was approved by the US FDA [22]. A phase 3 clinical trial of the Ixchiq (VLA1553) vaccine, involving 4115 healthy participants in the USA, found that after a single injection, 98.9% of vaccinated individuals developed CHIKV-specific neutralizing antibodies [23]. Live attenuated viruses show limited replication in humans and can induce a good immune response, but their safety profile is often suboptimal. Therefore, it is necessary to conduct research on highly safe recombinant proteins or nucleic acid vaccines to expand the scope of vaccination targets.

In this study, we sought to evaluate the immunogenicity of several CHIKV E protein-based DNA vaccines in mice, determine the correlation between CHIKV E protein glycosylation and immunogenicity, and identify important considerations for vaccine design. The envelope proteins of various human pathogens, including HIV-1, influenza virus, Lassa virus, SARS, Zika virus, Dengue virus, and Ebola virus, have evolved to be extensively glycosylated. These glycans, derived from host cells, play diverse structural and functional roles throughout the viral life cycle, ranging from immune evasion via glycan shielding to the enhancement of immune cell infection [24].

The results of the present study revealed that a CHIKV DNA vaccine based on the pVax1 vector induced antigen-specific immune responses in mice. The total IgG titers in the groups immunized with CHIKV E protein were higher than that of the groups immunized with the EΔgln, EI/II, or EI/IIΔgln vaccines (Figure 2A). Moreover, the group immunized with the CHIKV E protein vaccine showed significantly higher NAb titers than the control group (Figure 2B). However, the cell-mediated immune responses in the group immunized with the E1/2Δgln vaccine were stronger than those in the other groups (Figure 3). The multiplex immune responses in the group immunized with the E1/2Δgln vaccine were also higher than those in the other groups (Figure 4 and Figure 5). Conflicting results were confirmed upon analyzing the humoral and T-cell immune responses, which were thought to be due to increased stability of the wild-type CHIKV E protein due to the glycosylation of the antigen, thereby inducing high antibody titers. In addition, one possible explanation for the enhanced T cell responses observed with N-glycosylation-deficient vaccines is alterations in antigen presentation and processing. N-glycosylation has been implicated in protein folding, stability, and antigenicity, and its deficiency may lead to changes in the conformation or accessibility of antigenic epitopes, resulting in enhanced recognition by T cells. Moreover, the absence of N-glycosylation has the potential to promote the direct recognition of antigenic peptides by T cells, bypassing the need for processing and presentation by APCs. This may lead to a more robust and direct activation of T cell responses, contributing to the observed increase in T cell cytokine production and proliferation.

In previous studies supporting these findings, cell-specific glycoform distribution and glycan shielding analyses were performed on the glycosylated S protein, which has important implications for SARS-CoV-2 infection and immune responses. A novel SMG vaccine was designed to avoid glycan shielding and expose the epitope, thereby enhancing immune recognition [25]. This allows conserved epitopes to be more exposed to the immune system, leading to more effective and broadly protective T cell responses against the virus and its variants [26]. Another study found that mutations in glycosylation sites on the Ebola virus glycoprotein affect antigenicity and immunogenicity differently depending on the subunit; mutations in GP2 diminish both, possibly due to impaired dimerization, whereas mutations in GP1 near antibody epitopes may enhance immunogenicity by exposing the epitopes [27]. Furthermore, a study on the JEV DNA vaccine showed that immunization with DNA vaccines containing the N154A mutation enhances interleukin-4 secretion, IgG1 antibody induction, and anti-JEV antibody titers and provided complete protection against JEV challenge, suggesting the potential of this mutant as a DNA vaccine against JEV [28].

As a result, viral surface glycans may hinder the immune response by shielding conserved epitopes, which is crucial for universal vaccine development. Thus, removing glycosylation around these epitopes in nucleic acid vaccine design could be an effective strategy for universal vaccine development against viral pathogens [29].

## 5. Conclusions

In summary, the results of this study confirmed that the CHIKV DNA vaccine candidates induce humoral immune responses, while the non-glycosylated candidate induces a strong cell-mediated immune response. Nevertheless, it is essential to acknowledge the limitations of this study. Although our findings suggest a novel role for N-glycosylation in T cell immune regulation, further investigation is needed to elucidate the underlying mechanisms and confirm the generalizability of our observations. These includes confirming immunogenicity in animal models other than C57BL/6 mice and determining the specific effects of N-glycosylation deficiency on different T cell subsets, such as CD4^+^ and CD8^+^ T cells. Further studies are required to select an appropriate animal model to confirm the vaccine’s ability to prevent viral infections in vivo. These findings are useful for understanding the role of E protein glycosylation in CHIKV infection and vaccine development and as a reference for vaccine design and development against emerging pathogens.

## Data Availability

Data used in all analyses are included in this article. All other inquiries can be directed to the corresponding authors.
